# 625. SPR206 Pharmacokinetics (PK) in Plasma, Epithelial Lining Fluid (ELF), and Alveolar Macrophages (AM) in Healthy Adult Subjects

**DOI:** 10.1093/ofid/ofac492.677

**Published:** 2022-12-15

**Authors:** Keith A Rodvold, Justin Bader, Vipul K Gupta, Troy Lister, Praveen Srivastava, Jon Bruss

**Affiliations:** University of Illinois Chicago, Chicago, Illinois; Spero Therapeutics, Cambridge, Massachusetts; Spero Therapeutics, Cambridge, Massachusetts; Spero Therapeutics, Cambridge, Massachusetts; Spero Therapeutics, Cambridge, Massachusetts; Spero Therapeutics, Cambridge, Massachusetts

## Abstract

**Background:**

SPR206 is a novel intravenously (IV)-administered next-generation polymyxin being developed for the treatment of multi-drug resistant (MDR) Gram-negative (GN) infections. A Phase 1 bronchoalveolar lavage (BAL) study was conducted to evaluate SPR206 safety and PK in plasma and pulmonary matrices (ELF and AM) in healthy volunteers.

**Methods:**

Subjects received 100 mg SPR206 administered intravenously over 1 h q8h for 3 consecutive doses. Blood samples were collected pre-dose (second and third dose) and at 2, 3, 4, 6, and 8 h after the start of the last dose. Each subject underwent one standardized bronchoscopy with BAL at 2, 3, 4, 6 or 8 hours after the start of the third IV infusion. Concentrations of SPR206 in plasma, BAL, and cell pellet were measured with a validated LC-MS/MS assay. Plasma, ELF, and AM PK parameters were determined by noncompartmental analysis. SPR206 AUC_0–8_ in ELF and AM were calculated using the mean concentration values at the BAL sampling times. SPR206 AUC_0–8_ ratios of ELF and AM to unbound plasma were determined using plasma protein binding value of 8.6%.

**Results:**

Thirty-four subjects were enrolled and completed the study and 30 of 34 completed bronchoscopy. The mean SPR206 C_max_ concentrations in plasma, ELF, and AM were 4395.0, 735.5, and 860.6 ng/mL, respectively. Mean AUC_0-8 h_ values in plasma, ELF, and AM concentrations were 20120.7, 4859.8, and 6026.4 ng*h/mL, respectively--leading to a *free* ELF to plasma concentration ratio of 0.264 and *free* AM to plasma ratio of 0.328. Importantly, mean concentrations of SPR206 in the ELF achieved lung exposures above its minimum inhibitory concentration for the targeted gram-negative pathogens for the entire 8-hour dosing interval. Figure 1 displays the mean (±SD) SPR206 total and unbound plasma, ELF, and AM concentrations at the BAL sampling times. Overall, SPR206 was well tolerated; 22 subjects (64.7%) experienced at least 1 TEAE, mostly mild with a few moderate. The most frequently reported TEAEs were oral paresthesia [10 subjects (29.4%)] and nausea [2 subjects (5.9%)], which were reversible.

**Conclusion:**

This study demonstrates pulmonary penetration of SPR206 and supports further development of SPR206 for the treatment of patients with pneumonia caused by MDR GN infections.

**Figure 1.**
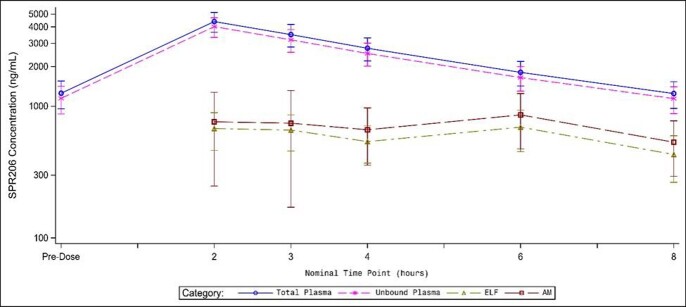
Mean (±SD) plasma (total and unbound), epithelial lining fluid (ELF), and alveolar macrophage (AM) concentrations of SPR206 in healthy adult subjects. This study was conducted in collaboration with, and with financial support from, the United States Department of Defense (Award No. W81XWH1910295).

**Disclosures:**

**Keith A. Rodvold, PharmD**, Spero Therapeutics: Advisor/Consultant|Spero Therapeutics: Honoraria **Justin Bader, n/a**, Spero Therapeutics: Employee **Vipul K. Gupta, Ph.D.**, Spero Therapeutics: Employee **Troy Lister, Ph.D.**, Spero Therapeutics: Employee **Praveen Srivastava, Ph.D.**, Spero Therapeutics: Employee **Jon Bruss, M.D.**, Spero Therapeutics: Employee.

